# Eliminating the influence of head and eye movements on the estimation of tear film surface parameters in lateral shearing interferometry

**DOI:** 10.1364/BOE.559750

**Published:** 2025-04-09

**Authors:** Dorota H. Szczesna-Iskander, D. Robert Iskander

**Affiliations:** 1 Department of Optics and Photonics, Wroclaw University of Science and Technology, Wybrzeze Wyspianskiego 27, 50–370 Wroclaw, Poland; 2 Department of Biomedical Engineering, Wroclaw University of Science and Technology, Wroclaw, Poland

## Abstract

Lateral sharing interferometry (LSI) can be used to assess tear film surface quality (TFSQ). Current estimates of TFSQ parameters are based on spectral measures of interferograms and are therefore dependent on the head and eye movements (HEM), especially those of longitudinal character. We propose an approach to address this in which the cross-ambiguity function is used to estimate the apparent change in the spatial scale robustly. Further, this estimate is used to scale the spectral representation of the interferogram, from which the TFSQ parameters are estimated. As a result, improved quality of spectral-based estimates of TFSQ has been achieved, where the effect of HEM is minimized. The adequacy of this approach has been confirmed with interferometry images of a moving glass sphere resembling a cornea surface. The results of the analysis for real eyes acquisitions are discussed in the context of the proposed spectral scaling, which provides new and important insight into the LSI-based assessment of TFSQ. The proposed method is more general and can be readily adapted to other types of interferometry or profilometry measurements of moving objects.

## 1. Introduction

Lateral shearing interferometry (LSI) is one of the most sensitive non-invasive methods for evaluating tear film surface quality (TFSQ) [1,2]. That high sensitivity is figuratively speaking an advantage in disguise, because when assessing tear film dynamics one has to deal with the ever-present head and eye movements (HEM). In a controlled laboratory environment, the influence of HEM can be reduced by using heavy headrests, bite bars, or head restraints. However, such measures are not applicable in clinical settings, leading to HEM preventing the widespread use of LSI technology in the diagnosis of dry eye disease (DED) [3].

Current state-of-the-art method of estimating TFSQ from LSI interferograms is based on combining traditional spatial spectrum estimation techniques with morphological image processing [4]. A spatial-average-localized weighted estimate of the first harmonic of the interference fringes—the so called parameter $M_{2}$ —was shown to be robust for transverse eye movements as well as for those of longitudinal character, occurring naturally [5], provided they are small in amplitude. However, in a typical clinical setting, particularly when subject’s head is placed in a standard headrest [6], HEM may substantially contribute to the recorded spatial frequency of the interferogram, consequently leading to biased estimates of spectral-based tear film surface parameters. In other words, assuming stable TFSQ, HEM cause an unwanted change in the spatial scale of the acquired interferograms that subsequently manifests itself in a change in the observed frequency of the interferogram, affecting interpretation of TFSQ dynamics.

Typically, the amplitudes of longitudinal eye movements, with spectral content associated with the cardio-pulmonary system, amount to 40−
50μ
 m [5]. In the context of LSI-based assessment of TFSQ, such amplitudes are considered small. The amplitudes of head displacement, when measured in a standard headrest setup, are typically in the range of ±
100μ
 m [6], and in LSI those amplitudes are considered moderate. Larger amplitudes of longitudinal eye movements predominantly occur in the post-blink phase as the eyeball moves forward from the previous retraction phase of about 1 mm [7], to resume its pre-blink position. Large amplitudes of HEM, substantially exceeding ±
100μ
 , predominately occur in naïve subjects with heavy breathing or in those showing some anxiety during the recordings [8].

Focusing on the assessment on tear film homeostasis in the tear film dynamics was proposed to be an alternative way for diagnosing DED [9,10]. Just after a blink, the upward movement of tear film is observed, theoretically driven by the unequal distribution of lipids following the blink or both lipid and aqueous layers of tear film [11]. Further, the post-blink spreading of the tear film is influenced by its quality, stability and viscosity [12]. A clinically important parameter of tear film leveling is being evaluated at this phase [13,14].

There is a shortage of studies on post-blink tear film behavior because the dynamics of the tear film between the blinks is difficult to assess in a clinical setting. Moreover, instrumentation that employs low sampling frequency rates is unable to accurately resolve this important phase of tear film behavior [15]. Contrarily, LSI offers solutions to fill this gap. Nevertheless, the estimates of spectral-based TFSQ parameters from LSI are influenced by HEM and it is the aim of this study to devise an algorithm that would reduce this effect.

## 2. Methods

### 2.1. Preliminaries

**Fig. 1.**
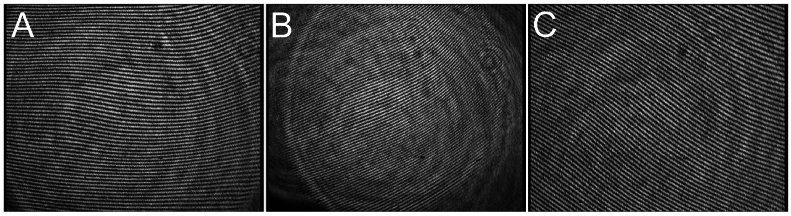
Illustrative interferograms of a glass sphere placed at the focal point of the LSI instrument (A), farther away ( −
100μ
m ) from the focal point (B) and closer to the instrument ( +100μ
m ) than its focal point (C). An apparent change if the interferogram frequency appears.

Consider the case of a stationary rigid surface (e.g., a glass sphere resembling corneal surface) placed in front of the LSI instrument, so its center of curvature is at the focal point. The spherical wavefront exiting the instrument illuminates the area of about 5 to 6 mm in diameter, reflects, and comes back on the same path to the instrument. As a result, an interferogram such as that shown in Fig. 1(A) is obtained. The shape and regularity of the interference fringes observed on the glass sphere are similar to those of tear film that is evenly spread over the cornea of a healthy eye. The fundamental frequency (first harmonic) of this interferogram can be readily estimated from the Fourier spectrum of the interferogram. Let then position that rigid surface at a plane that is farther away from the focal point (Fig. 1(B)) or at a plane that is closer to the instrument than the focal point (Fig. 1(C)). Apparent changes in the estimated fundamental frequency are then observed and, as a consequence, those changes influence the estimates of $M_{2}$ , as it was shown earlier [16]. The problem is exacerbated because the spatial resolution of such interferogram is low due to the use of a CCD camera and there is a spectral leakage present that cannot be effectively overcome by windowing methods (neither in spatial nor in the spectral domain).

The changes in frequency should be interpreted (disregarding any aberrations in the optical path) as spatial scaling of the signal, for which the following pair of Fourier transforms is relevant

(1) $$s(ax)⟷\frac{1}{|a|}S\left(\frac{f}{a}\right),\;a\neq 0.$$

where s(x) and S(f) is the spatial interferometry signal and its spectral representation, respectively. When the parameter a≈
1 , the scaling of the spectral content is not warranted. However, in the case of substantial object’s displacement from a focal point, such a scaling procedure needs to be implemented, so that changes in object’s position are not subsequently interpreted as changes in the surface quality. To realize this in two-dimensional interferograms acquired as digital images, the spatial frequency domain has to be appropriately scaled as well as the integration operation, used for estimating $M_{2}$ , has to be appropriately realized [16]. However, before such a scaling of the frequency spectrum can be undertaken, a robust estimator for the scaling parameter a needs to be considered.

### 2.2. Estimating the scale parameter a

Consider then the case of a one-dimensional sinusoidal signal

s(x)=Asin⁡
(2π
fx+ϕ
)

and its scaled version

sa(x)=Aasin⁡
(2π
afx+ϕ
a),

with arbitrary scales ( A , $A_{a}$ ) and phases ( ϕ
 , ϕ
a ), both embedded in white Gaussian noise at a given SNR (signal-to-noise ratio). The spectral representations of these signals will clearly indicate frequency f and af , provided that the SNR is high. However, for low SNR and particularly that below 0 dB, those estimates will no longer be reliable. Consider further the cross ambiguity function, defined as

**Fig. 2.**
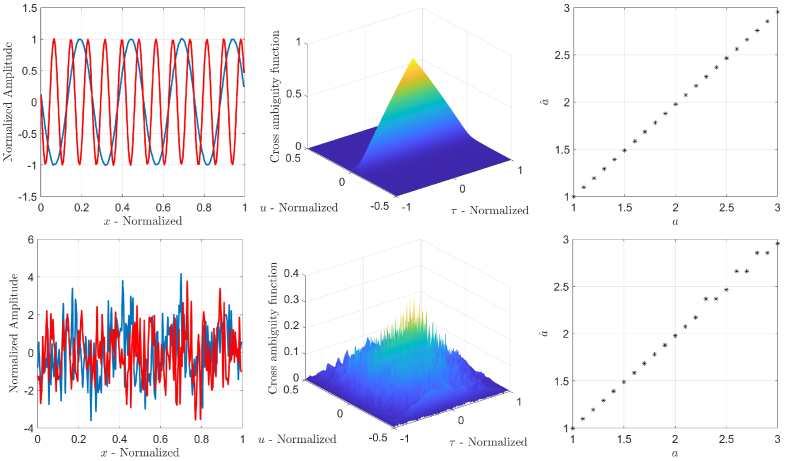
An illustrative example of robust estimation of the scale parameter a for two sinusoidal signals for SNR =40 dB (top row) and SNR =−
5 dB (bottom row).

S(τ
,u)=∫
−
∞
∞
s˜(x)s˜a∗
(x−
τ
)e−
j2π
uxdx

where $\tilde{s}(x)$ and ${\tilde{s}}_{a}(x)$ are the analytic (complex) equivalents of s(x) and $s_{a}(x)$ , respectively, obtained via Hilbert transformation and ^*^ denotes conjugation. A robust estimator $\hat{a}$ of the scale parameter a is obtained from $u_{\left(\max\right)}$ , for which

$$\int_{-\infty }^{\infty }S(\tau ,u)d\tau$$

is maximized. Figure 2 shows an illustrative numerical example of two sinusoidal signals spatially scaled by a , that ranges from 1 to 3 in steps of 0.1, for two SNR values: 40 dB (top row) and −
5 dB (bottom row). The plots and surfs in the first and the second column of Fig. 2 are for the case when a=3 . It is worth noting that the evident robustness of the cross ambiguity function based scale estimator does not depend on the amplitudes and phases of the original signal and its scaled version.

### 2.3. Implementing the spectral scaling

Having the scale estimate $\hat{a}$ between a given interferogram and the reference (REF) interferogram one is able, using Eq. (1), to adequately scale the Fourier spectrum and derive a "displacement-free" estimator of $M_{2}$ , defined as

**Fig. 3.**
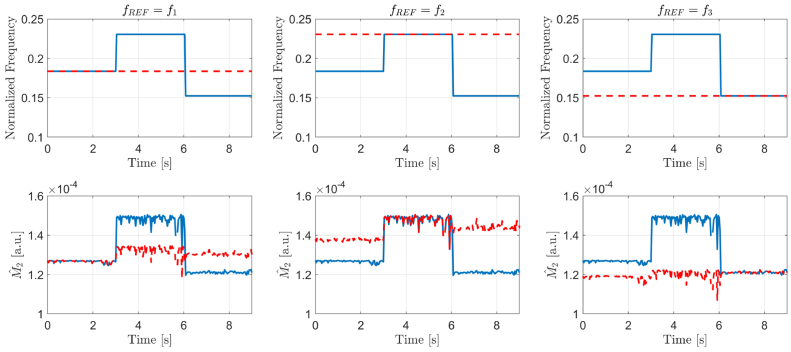
Results for the glass sphere object placed at three different positions with respect to the LSI instrument. Estimates of the first harmonic frequencies (top row) and those of the parameter $M_{2}$ (bottom row) for unscaled (blue line) and scaled (red dashed line) interferogram signals. Columns indicate which object position is considered as a reference.

M2=∫
f0−
Δ
f/2f0+Δ
f/2W(f)S(f)df,

where $f_{0}$ is the fundamental frequency of the interferogram, Δ
f is empirically set to 0.15 in the normalized frequency scale and W(f) is a suitable weighting window. In the discrete case, the estimator ${\hat{M}}_{2}$ of $M_{2}$ is realized via trapezoidal integration with the maximum likelihood estimator ${\hat{f}}_{0}$ of $f_{0}$ . Figure 3 shows the result of applying the proposed procedure to a concatenated video of three separate 3-second interferometric sequences for a rigid surface placed at the focal point, away from it, and closer to the instrument than the focal point, respectively. The sampling rate for acquiring the LSI video sequences is 25 Hz. The illustrative interferograms shown in Fig. 1 are representatives of those three sequences. The corresponding normalized fundamental frequencies for those three object positions have been estimated using Welch’s method at $f_{1}=0.18$ , $f_{2}=0.23$ and $f_{3}=0.15$ , respectively.

**Fig. 4.**
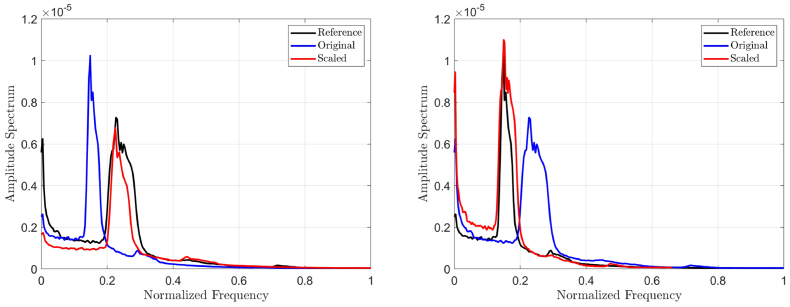
The effect of up-scaling (left) and down-scaling (right) the amplitude spectrum of the interferogram.

Although the variance of the frequency estimator across all given sequences is zero, that is not the case for the estimator ${\hat{M}}_{2}$ of the parameter $M_{2}$ , indicating that it is influenced by the environmental conditions and the ambient noise of the otherwise static measured object. Having three different object positions, three reference cases for scaling can be considered. In the left column of Fig. 3, the first frame of the video is set as a reference. Consequently, after rescaling, more consistent estimates of $M_{2}$ are being achieved. Similar results are being observed when the reference frame is set as one from the second (middle column of Fig. 3) or third (right column of Fig. 3) interferogram sequence. It is important to note that for estimating $M_{2}$ down-scaling ( $f_{REF}=f_{3}$ ) results in smaller variance of ${\hat{M}}_{2}$ than that achieved when interferograms are up-scaled ( $f_{REF}=f_{2}$ ). Figure 4 shows the effect of up-scaling the amplitude spectra of interferograms from the lowest fundamental frequency of $f_{3}=0.15$ to the highest fundamental frequency of $f_{2}=0.23$ , set as a reference (Fig. 4 (left)) as well as the effect of down-scaling of the spectral content from the highest fundamental frequency of $f_{2}=0.23$ to the lowest of $f_{3}=0.15$ , set as a reference (Fig. 4 (right)). For $f_{REF}=f_{3}$ , the correlation between the scaled ${\hat{M}}_{2}$ and the fundamental frequency becomes statistically insignificant ( r=−
0.315,p=0.117 ) compared to its original unscaled value ( r=0.794,p≪
0.001 ).

When considering interferometric sequence of TFSQ acquired in-vivo on eye, where the placement of the corneal surface with respect to the focal point of the instrument cannot be precisely controlled, the first step of analysis is the identification of a valid reference interferogram, for which the fundamental frequency is lowest within the recording.

## 3. Results

**Fig. 5.**
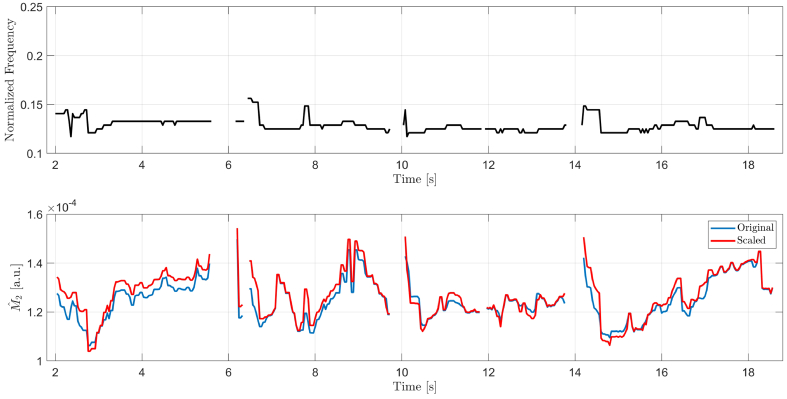
The case of negligible HEM. Results for an illustrative fragment of an in-vitro LSI recording of the human eye: estimated fundamental frequency of interferogram (top) and the corresponding estimates of $M_{2}$ (bottom) for unscaled (blue line) and scaled (red) spectra.

Two cases of LSI-based assessment of TFSQ for real eyes were considered. One where HEM are negligible and another one, where they are moderate. Figure 5 shows a fragment of LSI recording, in which four interblink intervals are acquired. The subject’s head is placed in a heavy headrest resulting in negligible HEM. The subject is not naïve to the measurement and understands the technology behind it. The estimates of the fundamental frequency are shown as a time-series in the top panel whereas the estimates of $M_{2}$ are given in the bottom panel and shown in blue and red for unscaled and scaled spectra, respectively. Scaling of spectra does not substantially change the estimates of $M_{2}$ . One may presume then that in such cases the scaling algorithm is not warranted.

**Fig. 6.**
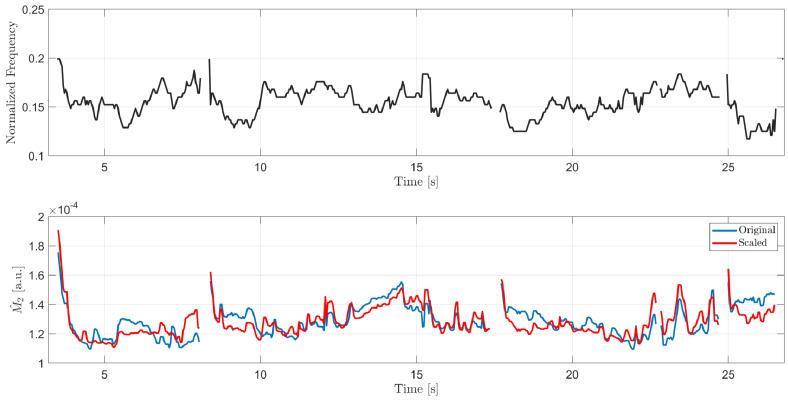
The case of moderate HEM. Results for an illustrative fragment of an in-vitro LSI recording of the human eye: estimated fundamental frequency of interferogram (top) and the corresponding estimates of $M_{2}$ (bottom) for unscaled (blue line) and scaled (red) spectra.

The second case is presented in Fig. 6 that shows a fragment of another LSI recording, also with four interblink intervals, but for a subject that is naïve to the measurement and where HEM are ranked as moderate. Qualitatively, the scaled version of ${\hat{M}}_{2}$ may not look much different from that of the unscaled one. However, for each interblink interval, the estimates of time at which TFSQ is the highest post blink (i.e., corresponding to local minimum of ${\hat{M}}_{2}$ ) may be substantially different. As a consequence interpretation of the results related, for example, to tear film leveling phase [14], may be altered. In this particular example, the estimates of tear film leveling time for each interblink, calculated from the unsmoothed representations of ${\hat{M}}_{2}$ time series, are {1.1,2.8,10.2,0.1} and {1.7,1.6,9.9,0.5} , for the unscaled and scaled version of ${\hat{M}}_{2}$ , respectively. The differences are proportionally larger for short interblink intervals, in which TFSQ is usually less stable.

The algorithm was also tested on recordings with large HEM and resulted in a failure to adequately estimate the change in spatial scale. Therefore such recordings are still deemed to be unusable.

## 4. Discussion

Various methods have been used to study the tear film dynamics time after blinking. The assessment of upward drift of the reflective light particles in the tear film showed their decay in time and that they stabilize after about 1 s [17]. The automatic tracking of the reflective light particles spreading at the sampling rate of 10 Hz showed consistency in blink cycles for the particular subject [9] and correlation between the average speed of the particles and tear film break-up time [10]. Using wavefront aberration, Ferrer-Blasco et al. [18] showed that it takes from five to six seconds for the tear film to reach its optimal optical efficiency. Differences were also demonstrated between eyes with DED and healthy eyes, with root-means square (RMS) values reaching a minimum after half the time in DED eyes. Goto and Tseng [19], using white light interferometry, found that in healthy eyes the mean lipid spreading time lasted about 0.3 s, but the lipid deficiency led to a mean spreading time of 3.5 s, and in those subjects with aqueous humor deficiency the mean spreading time was 2.2 s. Those varying results indicate that assessing TFSQ is challenging in a clinical setting and it depends on the utilized imaging instrumentation. The LSI technique is objective, noninvasive, and provides detailed information on tear film dynamics in natural, forced or suppressed blinking conditions at temporal sampling rates that can precisely resolve the post-blink phase of tear film leveling. Nevertheless, its sensitivity to HEM may sometimes be problematic.

An apparent limitation of the study is the lack of a comprehensive analysis of the clinical and statistical significance of the proposed method for eliminating the effect of HEM on the estimation of tear film parameters. Nevertheless, some information on this influence can already be obtained from the presented illustrative LSI recording of a naïve subject, in which the mean relative change in the tear film leveling time (across four interblink intervals) amounts to 48±
35%
 .

As noted earlier, the proposed method for eliminating the effect of HEM could not adequately estimate the spatial scale changes for large HEM. There are two main reasons for this phenomenon. First, when the center of curvature of the eye is placed much farther away from the LSI focal point, the spatial sampling of the imaging device is too small, resulting in aliasing. Second, when the center of curvature of the eye is placed too close to the instrument’s focal point, the resulting principal frequency of the interferogram is embedded in low frequency content of the image, producing poor frequency estimates. These are technical limitations that can be partially alleviated by incorporating a higher resolution CCD camera or changing the optical configuration to illuminate larger portion of corneal surface than in the current LSI, to increase the spatial frequency resolution.

The result of this study clearly showed that when head and eye movements are small, current method for assessing TFSQ with LSI is adequate. For moderate amplitudes of HEM, the proposed algorithm minimizes the influence of them and, hence, constitutes another step towards achieving clinically relevant acceptance of LSI as an effective method of TFSQ analysis.

## Data Availability

Data underlying the results presented in this paper are not publicly available at this time but may be obtained from the authors upon reasonable request.
